# Glucose-6-phosphate dehydrogenase deficiency in people living in malaria endemic districts of Nepal

**DOI:** 10.1186/s12936-017-1864-2

**Published:** 2017-05-23

**Authors:** Prakash Ghimire, Nihal Singh, Leonard Ortega, Komal Raj Rijal, Bipin Adhikari, Garib Das Thakur, Baburam Marasini

**Affiliations:** 1 World Health Organization, Country Office Nepal, UN House, Pulchowk, Lalitpur, Nepal; 2 Global Malaria Programme, World Health Organization, Geneva, Switzerland; 3 Central Department of Microbiology, Tribhuvan University, Kathmandu, Nepal; 4 Mahidol Oxford Research Unit, Bangkok, Thailand; 5 Ministry of Health, Kathmandu, Nepal; 6 Epidemiology & Disease Control Division, Ministry of Health, Kathmandu, Nepal

**Keywords:** Malaria, G6PDd, Nepal, Ethnic group, Antimalarial drugs, Risk

## Abstract

**Background:**

Glucose-6-phosphate dehydrogenase (G6PD) is a rate limiting enzyme of the pentose phosphate pathway and is closely associated with the haemolytic disorders among patients receiving anti-malarial drugs, such as primaquine. G6PD deficiency (G6PDd) is an impending factor for radical treatment of malaria which affects the clearance of gametocytes from the blood and subsequent delay in the achievement of malaria elimination. The main objective of this study was to assess the prevalence of G6PD deficiency in six malaria endemic districts in Southern Nepal.

**Methods:**

A cross-sectional population based prevalence survey was conducted in six malaria endemic districts of Nepal, during April–Dec 2013. A total of 1341 blood samples were tested for G6PDd using two different rapid diagnostic test kits (Binax-Now^®^ and Care Start™). Equal proportions of participants from each district (n ≥ 200) were enrolled considering ethnic and demographic representation of the population groups.

**Results:**

Out of total 1341 blood specimens collected from six districts, the overall prevalence of G6PDd was 97/1341; 7.23% on Binax Now and 81/1341; 6.0% on Care Start test. Higher prevalence was observed in male than females [Binax Now: male 10.2%; 53/521 versus female 5.4%; 44/820 (p = 0.003) and Care Start: male 8.4%; 44/521 versus female 4.5%; 37/820 (p = 0.003)]. G6PDd was higher in ethnic groups Rajbanshi (11.7%; 19/162) and Tharu (5.6%; 56/1005) (p = 0.006), major inhabitant of the endemic districts. Higher prevalence of G6PDd was found in Jhapa (22/224; 9.8%) and Morang districts (18/225; 8%) (p = 0.031). In a multivariate analysis, male were found at more risk for G6PDd than females, on Binax test (aOR = 1.97; CI 1.28–3.03; p = 0.002) and Care Start test (aOR = 1.86; CI 1.16–2.97; p = 0.009).

**Conclusions:**

The higher prevalence of G6PDd in certain ethnic group, gender and geographical region clearly demonstrates clustering of the cases and ascertained the risk groups within the population. This is the first study in Nepal which identified the vulnerable population groups for G6PDd in malaria endemic districts. The finding of this study warrants the need for G6PDd testing in vulnerable population groups in endemic districts, and also facilitates use of primaquine in mass supporting timely progress for malaria elimination.

## Background

More than 2.5 billion people are at risk of infection by the malaria parasite *Plasmodium vivax*, and each year more than a hundred million suffer from clinical infection [1]. Despite that vivax malaria had been established as a relatively benign malaria, studies from endemic areas and particularly in travelers to endemic areas, fatal and pernicious outcome has been reported [2, 3]. *Plasmodium vivax* can remain as dormant hypnozoite in liver cell, without clinical manifestations, and this is a major problem [4]. The only drug which can kill the hypnozoites is primaquine, which has been in continuous use since 1952. Nevertheless, at therapeutic dose against the hypnozoites, primaquine can cause a self-limiting to severe haemolytic anaemia in patients with an inborn deficiency of enzyme-G6PD [5, 6].

Deficiency of G6PD (G6PDd) is a genetic abnormality, one of the most prevalent polymorphisms and enzymopathies in humans, specifically, in males [7]. This genetic defect was discovered in 1956 after the development of haemolytic anaemia following the administration of the anti-malarial primaquine [8]. About 8% of the people who are exposed to malaria have an inherited disorder that impairs G6PD, leaving them vulnerable to develop clinical consequences (haemolytic anaemia). About 400 million people worldwide are estimated to be affected by G6PDd in malaria endemic regions [6].

More than 80% of vivax malaria attacks occur in South and Southeast Asia, where Mediterranean-like G6PDd dominates the other variants [7, 9]. Patients having below 30% of the normal G6PD activity are vulnerable to primaquine induced haemolysis. However, heterozygote females with higher mean red cell activities may still show substantial haemolysis [10].

Substantial haemolysis can be still seen in some heterozygote females who have intermediate G6PD activity and can have test as normal or not deficient in qualitative G6PD screening tests. Intermediate deficiency (30–80% of normal) and normal enzyme activity (>80% of normal) can be differentiated only with a quantitative test. While prescribing primaquine for 14 days to females who are considered to have intermediate G6PD activity, counselling on potential development of signs and symptoms of haemolytic anaemia is required [10].

G6PD-deficient erythrocytes are more susceptible to destruction by oxidative stress than normal erythrocytes due to the lower NADPH levels. Individuals with this genetic defect may exhibit non-immune haemolytic anaemia in response to a number of stimuli, most commonly, infections or exposure to certain medications or chemicals [11]. The geographical distribution (prevalence in general) of malaria closely resembles the global distribution of deficient G6PD variants [12]. It is postulated that increase in G6PDd has been associated with the natural selection of G6PD deficient variants which confers protection or resistance against malaria caused by *Plasmodium falciparum* and *Plasmodium* spp. [13].

In the context of malaria elimination, vector control measures, such as long-lasting insecticide-treated bed nets, indoor residual insecticide spraying along with prompt diagnosis and treatment of malaria infected patients are the most effective tools currently available [14]. Anti-malarial drugs are seen as crucial to eliminate malaria and the focus is on the role of drugs to block malaria transmission by killing gametocytes and reducing the pool of liver stage hypnozoites of *P. vivax* and *Plasmodium ovale* [15].

Nepal is making good progress in reducing the number of malaria cases significantly in last one decade. According to malaria risk micro-stratification 2013, malaria risk has dropped significantly among general population and reached to a level in which only 54 village development committees (VDC’s) are in high, 201 VDC’s in moderate and 999 VDC’s in low risk falling in 25 out of 75 districts of Nepal. The malaria risk population has reached to 48% (~13 million out of 27.7 million) of the country’s population, as compared to earlier 72% of the country’s population [16]. Since the malaria programme in Nepal is moving towards elimination, compliance in use of 14 days primaquine for confirmed *P. vivax* malaria cases without G6PD deficiency is recommended in the revised national malaria treatment protocol-2015 [17].

Therefore, it is necessary to determine the status of G6PDd in population living in malaria risk areas for the timely and successful elimination of malaria from the country. In order to generate the evidence for decision making on the need for G6PD testing in the health system, a cross-sectional study was under-taken to estimate the population prevalence of G6PDd in six malaria endemic districts of Nepal using immuno-chromatographic test kits: Binax-Now^®^ and Care-Start™test card.

## Methods

This cross-sectional prevalence study was conducted during April–December 2013. The study protocol was approved by the Nepal Health Research Council (NHRC) Ethical Review Board on 29 March 2013 (Reference No. 1134).

A total of 1341 volunteers from six selected districts were enrolled in the G6PDd prevalence study following estimation of sample size for the study. The six districts were selected based on the malaria prevalence and detection of suspected G6PDd cases by the existing health services in the region, known through national malaria programme reports. More than 200 volunteers in each district were enrolled and analysed in the study.

### Study sites

The study sites were malaria endemic village development committees (VDCs) of Jhapa, Morang, Dhanusha, Chitwan, Dang and Kailali districts, high malaria risk areas identified by the last 5 years malaria data and the recent micro-stratification study report [18].

### Inclusion and exclusion criteria

Volunteers who were between 5 and 60 years, without known chronic diseases, and consenting/assenting for voluntary participation were only enrolled for the study. Human subjects below 5 years of age and above 60 years, pregnant or lactating mothers, persons with chronic diseases, and persons having no written consent, were excluded from the study.

### Sample collection and test performance

In each district, malaria risk VDC’s and risk population were identified based on the district health records in coordination with district public health office. Informed written consent was received from all individuals before enrolling into the study. Study benefits and procedures were well explained before the written consent was obtained from each participant. The blood specimens were collected from enrolled volunteers.

Results for each patient (according to the qualitative G6PDd activity test) were provided to the volunteer participants, a consolidated report of the VDC was provided to staffs of the respective health facilities and a consolidated report of the district was provided to district health office (vector borne disease focal persons). G6PDd tests were performed during morning (6–10 a.m.) and evening (5–7 p.m.) avoiding high summer temperature in the districts and maintaining the temperature requirements (18–25 °C) for the test kits.

Whole blood specimens from the enrolled volunteer participants (2 and 10 µl) were collected and tested immediately using Care-Start™ [21] and Binax-NOW^®^ G6PDd test kits [22] in the field settings, following manufacturers’ instructions.

### Care-Start™ G6PD deficiency screening test

The Care-Start™ G6PD deficiency screening test contain the test strip encased in a flat plastic cassette (containing a buffer well, a sample well and a result window), a sample pipette, the assay buffer, an alcohol pad and a blood lancet. This RDT format is a qualitative enzyme chromatographic test, based on the reduction of colourless nitroblue tetrazolium dye to dark coloured formazan. Following the manufacturer instructions, 2 µl of blood was added into the sample well and two drops of buffer into the buffer well and allowed to laterally flow the samples by the buffer in the device. Test results were read visually after 10 min. Samples with normal G6PD activity produced a distinct purple colour in the result window, while no colour change was observed for samples with G6PDd subjects [19].

### Binax-NOW^®^ G6PD test

The BinaxNOW^®^ G6PD test is a qualitative enzyme chromatographic test (ECT) for detecting G6PD activity. The test device contains a lateral flow test strip comprised of a white sample pad and a reaction pad. The reaction pad contains the reagents necessary for the G6PD enzymatic reaction and the subsequent reduction of a nitroblue tetrazolium dye into its concomitant blue formazan product. When no change in the red colour of the sample front was observed at the test read time, the sample was presumed to be deficient in G6PD enzyme activity. Samples with normal G6PD activity produced a distinct colour change: the red sample colour changed to a brown/black colour on the upper half of the reaction pad [20].

### Statistical analysis

All demographic data of the volunteers (age, sex, ethnic group and place of residence) and G6PDd test results were recorded in Microsoft Excel and analysed using SPSS (IBM SPSS statistics for Windows for version 22.0). Descriptive and inferential statistics to ascertain the association between independent variables and dependent variable (BinaxNow test and Care-Start test) were conducted using Chi square test. All variables were entered into binary logistic model to evaluate the adjusted odds ratio. Statistical significance was set at p value less than 0.05.

## Results

### Demographic characteristics of participants

**Fig. 1:**
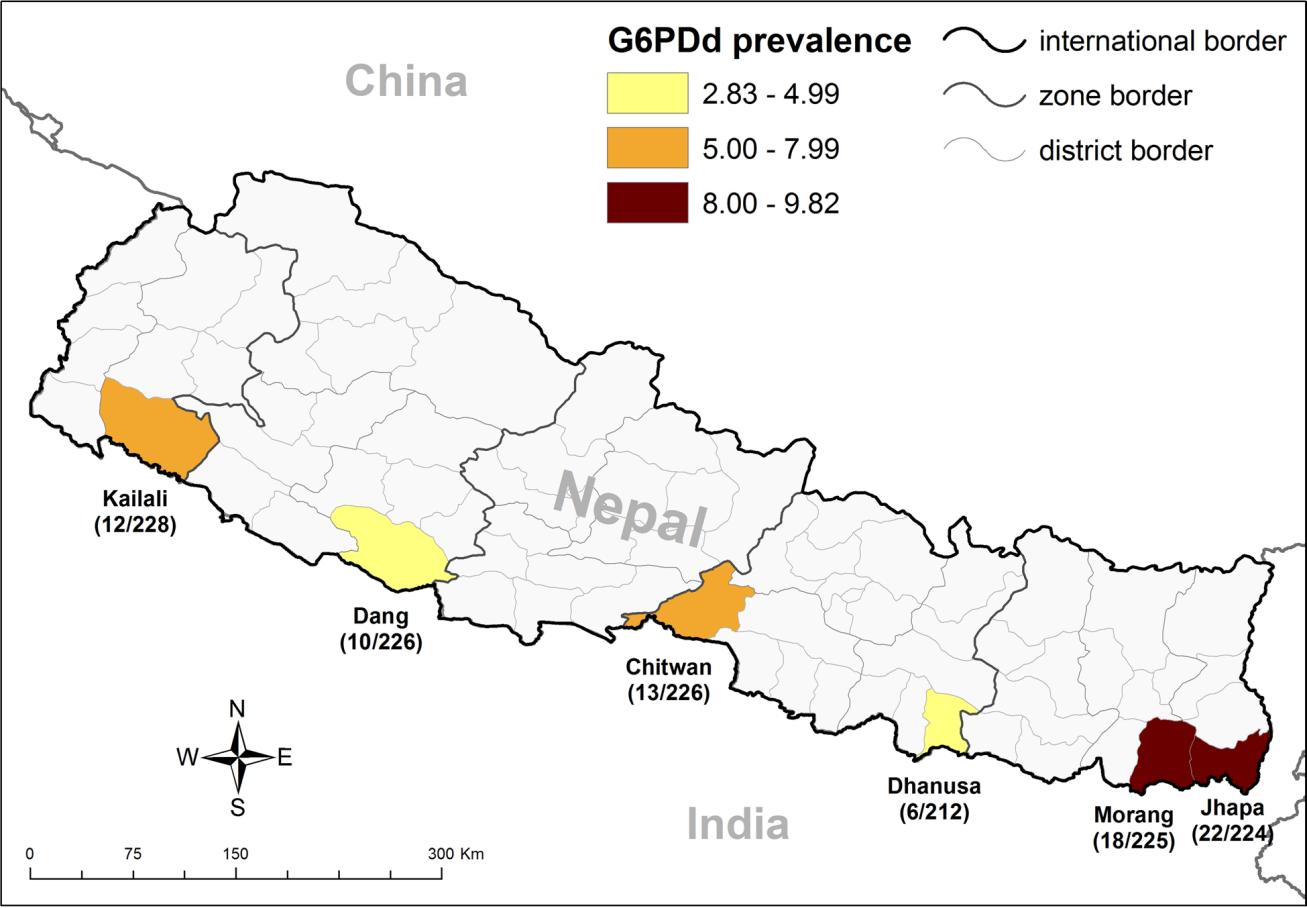
G6PD deficiency prevalence in Nepal-2013

Total of 1341 volunteer participant’s blood specimen were collected from six districts in malaria endemic region of Nepal (Fig. 1). Among total participants, 820 (61.1%) were male. Highest number of participants were from Tharu ethnic group (n = 1005). Among all participants, slightly more than half of the participants (51.2%) were from age less than 20 years. Number of samples in this study varied between the districts (212; 15.8% in Dhanusha) to (228; 17% in Kailali).

### Demographic characteristics in Binax test result

On BinaxNOW ^®^ G6PD test, higher G6PD deficiency was found associated with male (53/1341; 10.2%) compared to female (44/820; 5.4%) (p = 0.001) (Table 1). On logistic regression analysis (Table 2), male were found to be at significant risk (aOR = 1.97; CI 1.28–3.03) for G6PD deficiency (p = 0.002).

**Table 1:** Socio-demographic factors in relation to G6PD deficiency by Binax (n = 1341)

Characteristics	Number (%)	Binax result		p value
		Normal	Low	
		Number (%)	Number (%)	
Gender				
Female	820 (61.1)	776 (94.6)	44 (5.4)	0.001*
Male	521 (38.9)	468 (89.8)	53 (10.2)	
Ethnicity				
Rajbanshi	162 (12.1)	147 (90.7)	15 (9.3)	0.289
Brahmin and Chhetri	70 (5.2)	67 (95.7)	3 (4.3)	
Tharu	1005 (74.9)	930 (92.5)	75 (7.5)	
Others	104 (7.8)	100 (96.2)	4 (3.8)	
Age group (years)				
≤20	687 (51.2)	625 (91.0)	62 (9.0)	0.063
21–35	356 (26.5)	338 (94.9)	18 (5.1)	
36–50	203 (15.1)	190 (93.6)	13 (6.4)	
≥51	95 (7.1)	91 (95.8)	4 (4.2)	
Districts				
Dhanusa	212 (15.8)	203 (95.8)	9 (4.2)	0.501
Dang	226 (16.9)	205 (90.7)	21 (9.3)	
Kailali	228 (17)	211 (92.5)	17 (7.5)	
Chitwan	226 (16.9)	209 (92.5)	17 (7.5)	
Morang	225 (16.8)	209 (92.9)	16 (7.1)	
Jhapa	224 (16.7)	207 (92.4)	17 (7.6)	

* Significant by Chi Square test

**Table 2:** Multivariate logistic regression analysis for risk to G6PD deficiency using Binax (n = 1341)

Characteristics	Adjusted	95% CI	p value
	Odds ratio	OR-range	
District			
Dhanusha	0.6	0.13–2.77	0.51
Dang	1.52	0.35–6.59	0.57
Kailali	1.08	0.24–4.86	0.91
Chitwan	1.18	0.26–5.31	0.82
Morang	1.07	0.25–4.55	0.91
Ethnicity			
Rajbanshi	2.3	0.62–8.45	0.2
Brahmin and Chhetri	1.02	0.20–5.20	0.98
Tharu	1.93	0.54–6.87	0.31
Age group (years)			
<20	3.88	0.34–44.29	0.27
21–35	1.86	0.30–11.64	0.5
36–50	1.89	0.50–7.12	0.34
Age	1.01	0.96–1.06	0.55
Sex			
Male	1.97	1.28–3.03	0.002*

* Significant p value <0.05

### Demographic characteristics in Care-Start test result

On Care-Start test, higher G6PD deficiency was found to be associated with male (44/521; 8.4%) compared to female (37/820; 4.5%) (p = 0.003). On most vulnerable ethnic group analysis, it was found that Rajbanshi (19/162; 11.7%) ethnic group (p = 0.006) and few districts (Jhapa-22/224; 9.8% and Morang-18/225; 8%) were found to be associated with G6PD deficiency (Table 3). On further analysis (Table 4), consistent with BinaxNow test, male were found to be at higher risk (aOR = 1.86; CI 1.16–2.97) for G6PD deficiency (p = 0.009).

**Table 3:** Socio-demographic factors in relation to G6PD deficiency by Care Start (n = 1341)

Characteristics	Number (%)	Care Start result		p value
		Normal	Low	
		Number (%)	Number (%)	
Gender				
Female	820 (61.1)	783 (95.5)	37 (4.5)	0.003*
Male	521 (38.9)	477 (91.6)	44 (8.4)	
Ethnicity				
Rajbanshi	162 (12.1)	143 (88.3)	19 (11.7)	0.006*
Brahmin and Chhetri	70 (5.2)	69 (98.6)	1 (1.4)	
Tharu	1005 (74.9)	949 (94.4)	56 (5.6)	
Others	104 (7.8)	99 (95.2)	5 (4.8)	
Age group (years)				
≤20	687 (51.2)	639 (93.0)	48 (7.0)	0.448
21–35	356 (26.5)	339 (95.2)	17 (4.8)	
36–50	203 (15.1)	191 (94.1)	12 (5.9)	
≥51	95 (7.1)	91 (95.8)	4 (4.2)	
Districts				
Dhanusa	212 (15.8)	206 (97.2)	6 (2.8)	0.031*
Dang	226 (16.9)	216 (95.6)	10 (4.4)	
Kailali	228 (17)	216 (94.7)	12 (5.3)	
Chitwan	226 (16.9)	213 (94.2)	13 (5.8)	
Morang	225 (16.8)	207 (92.0)	18 (8.0)	
Jhapa	224 (16.7)	202 (90.2)	22 (9.8)	

* Significant by Chi square test

**Table 4:** Multivariate logistic regression analysis for risk to G6PD deficiency using Care Start (n = 1341)

Characteristics	Adjusted	95% CI	p value
	Odds ratio	OR-range	
District			
Dhanusha	0.28	0.058–1.36	0.11
Dang	0.49	0.10–2.31	0.37
Kailali	0.56	0.12–2.62	0.46
Chitwan	0.67	0.14–3.11	0.61
Morang	0.88	0.21–3.68	0.86
Ethnicity			
Rajbanshi	1.87	0.62–5.63	0.26
Brahmin and Chhetri	0.31	0.03–3.02	0.31
Tharu	1.61	0.41–6.23	0.49
Age group (years)			
<20	0.85	0.06–11.98	0.9
21–35	0.78	0.11–5.55	0.811
36–50	1.32	0.33–5.24	0.68
Age	0.98	0.93–1.04	0.67
Sex			
Male	1.86	1.16–2.97	0.009*

* Significant p value <0.05

### Operational characteristics of BinaxNOW ^®^ and Care-Start™ G6PDd rapid diagnostic tests

The researchers experience in using both the tests were as follows:

1. *Kit content* Care-Start kit was available with required accessories including lancets, alcohol swabs and droppers, while BinaxNow was deprived of adjunctive accessories, thus buying those additional accessories may not have been standardized for the kit.
2. *Processing steps* Care-Start kits were designed for one step, with all available peripherals, while BinaxNow had multiple steps which included the additional step of sample mixing with buffer in the supplied tube.
3. *Temperature of storage* Care-Start allowed the researchers to store and test up to 30 °C, which was bit flexible in temperate region in comparison to BinaxNow with the recommendation of its use below 25 °C.
4. *Visibility and interpretation of the test results* The result window of Care-Start test was clearly visible with distinct purple colour appearance for normal cases and transparent (white background) in G6PD deficient cases. In BinaxNow test, brown line appeared at the end of the test window for normal cases while for the deficient cases, the original red colour of the blood did not change in test window. The colour distinction in BinaxNow test is sometimes difficult due to proximity between red and brown colour backgrounds. In contrast, the colour change in Care Start kit is clearer as it is entirely different between normal and deficient subjects.
5. *Cost of the test kits* The procurement cost for each Care-Start test is around US$2 while it is almost double in case of BinaxNow, which could be a major barrier for malaria elimination programme.

## Discussion

Over one-third of the world’s population lives at risk of *P. vivax* infection [21]. Limited evidence underpins estimation of clinical cases, however, globally about 400 million clinical cases are reported annually [22], including potentially severe illness and death [23]. In the context of malaria elimination, therapy must target all infections, including asymptomatic and submicroscopic blood-stage infections, dormant liver-stage hypnozoites as well as clinical cases [24]. One of the many consequences of neglect for last half century of *P. vivax* has been the failure to address the primaquine toxicity problem with G6PD. No non-toxic therapeutic alternatives exist, and existing G6PDd diagnostics are largely impractical in point-of-care settings [25].

One of the recent studies conducted in Afghanistan, Bangladesh, Bhutan, India, Nepal, and Pakistan found that the G6PDd prevalence ranges from 3.8 to 15%, with regional “hot spots” exceeding 22%. It has also recommended to institute a human-centered design (HCD) approach of newborn screening which could build the evidence to translate the complex biology of G6PD deficiency and the bio-design of affordable technologies for detection and characterization of the G6PDd [26].

Studies so far have shown that Care-Start test has the higher sensitivity (Care-Start test = 100% and BinaxNow test = 54.5%) but lower specificity (Care-Start test = 72.1% and BinaxNow test = 100%) compared to BinaxNow test [27, 28].

In this study, prevalence of G6PD deficiency showed difference between males and females. In male, the prevalence of G6PDd remain higher in both the test kits [10.2% (aOR = 1.97; CI 1.28–2.03; p = 0.002) on BinaxNow and 8.4% (aOR = 1.86; CI 1.16–2.97; p = 0.009) on Care-Start]. The male preponderance of G6PD deficiency can be attributed to the X-linked inheritance in G6PD gene [29]. The higher prevalence of G6PDd in male is consistent with other studies conducted in Afghanistan [30] and Solomon Islands [31]. In Afghanistan, considerable difference in prevalence was found between males (10%) and females (2%) [30]. Similar study in Solomon Islands found the G6PD deficiency of 10.9% in male and 3.6% in female [31].

This study is the first study of its kind in Nepal to estimate G6PD deficiency prevalence in different ethnic groups within population, particularly residing in malaria endemic areas of Nepal. In India, G6PD deficiency was first reported by Baxi et al. in 1963 and the prevalence rate varied from 0 to 27% in different caste, ethnic, and linguistic groups [32].

In this study, the highest G6PD deficiency prevalence was detected in Rajbanshi ethnic group (19/162; 11.7%) on Care Start test (p = 0.006). Rajbanshi is one of the main ethnic group inhabitants of the Terai (malaria endemic regions for centuries) believed to have an innate resistance to malaria. Following a successful control of malaria through IRS (Insecticide Residual Spray) in early 1960s by National Malaria Eradication Office (NMEO), a large and heterogeneous non-Rajbanshi population has inhabited the area in recent years. By analysing NMEO records, prevalence of residual malaria cases has nearly been seven times lower among Rajbanshi compared to sympatric non-Rajbanshi [33]. This might be due to genetic resistance to malaria in the Rajbanshi ethnic groups.

Most of the districts in this study are bordering India, and ethnic groups such as Rajbanshi, Tharu, Satar, Dhimal and Mushar are the major inhabitants in those districts. In Nepal, status of G6PD deficiency in these ethnic groups is not clearly understood, although a few local studies have indicated some prevalence in the mentioned districts and populations. In some studies, hereditary anaemia such as sickle cell blood and glucose-6-phosphate dehydrogenase (G6PD) deficiency were found at a high rate among the Rajbanshi people [34, 35].

The current study findings showed that G6PDd is more prevalent in Rajbanshi ethnic groups compared to others by enzymatic chromatography method. The findings of this study are consistent with the findings from India where G6PDd prevalence ranged between 2.3 and 27% [36]. In another study, higher prevalence of G6PDd (27.5%) was found in one particular community (Vataliya Prajapati community) in Western India and 27.1% in Angami Nagas, a tribal group in Northeastern India [37]. Current study findings have been consistent with the clustering prevalence of G6PDd among different ethnic groups in India [36, 37].

The prevalence of G6PD deficiency varied between the endemic districts in this study. Jhapa (9.8%) and Morang (8%) showed the highest prevalence of G6PDd among six districts (p = 0.031). The heterogeneity of G6PDd in this study can be explained in part by the presence of Rajbanshi ethnic population in these districts. In addition, Rajbanshi ethnic groups have been identified as one of the vulnerable groups as they are linked with the marriage within the siblings of the same family.

More than 80% of vivax malaria with predomination of G6PD Mediterranean type occurs in Southeast Asia. Person having red cell G6PD activity <30% of the normal mean has G6PDd and such person experience haemolysis after administration of primaquine. Heterozygote females with higher mean red cell activities may still show haemolysis. As Care-Start and BinaxNow test kit are qualitative G6PDd screening test, they may still miss to detect intermediate G6PD activity in some of heterozygote females. This is one of major limitation of the study [7, 9, 10]. However, the study findings clearly showed more deficient males than females, which is a positive indication for using RDTs in these settings. In one of the study carried out in 2003, using blood samples from Nepalese population, showed that two cases of Mediterranean-type G6PDd, not India–Pakistan sub-type but Mediterranean-Middle East sub-type have been detected [38].

Most of the phenotypic tests detect G6PDd only in hemizygous male & homozygous female [39]. The cytochemical assay is the most reliable method to detect G6PDd in hemizygous, homozygous and heterozygous deficient individuals [40]. It is clear from available data that G6PDd in heterozygous women cannot be accurately identified through G6PD enzyme activity assays. The performance of qualitative G6PD tests might depend on the boundary or the cut-off point between normal and deficient, different variants type and geographical settings of the study sites. Temperature is one of the crucial factors that affect the test result. In case of BinaxNow test, if the test is performed at 37 °C, the deficient sample test results looks similar to that of normal sample at low temperature [41]. Practically, Care-Start test is field-friendly as it is operationally cost-effective, easy to perform and temperature flexible. Further studies on genetic variant of G6PD deficiency can be helpful in explaining the homo/heterogeneity of G6PDd in Nepalese population.

## Conclusions

Prevalence of G6PD deficiency in Nepalese population varies in ethnic groups, strongly recommending need of G6PDd testing before the start of primaquine for each confirmed *P. vivax* cases. Knowing the G6PDd status gives leverage to use 14 days primaquine in G6PD normal patients, while weekly primaquine under close clinical monitoring/medical supervision with ready access to blood transfusion services in G6PD deficient cases. In absence of appropriate G6PDd testing facilities and lack of knowledge on the prevalent genotypes and severity, it will be prudent to err on the safe side. Rational use of such radical treatment facilitates the country in moving forward for timely malaria elimination.
